# Lateral mini-incision via cervical midline approach versus traditional incision for unilateral thyroid cancer: a retrospective cohort study

**DOI:** 10.3389/fsurg.2026.1757544

**Published:** 2026-07-10

**Authors:** Wu Xiao, Wei Chen, Yong Yang, Lianghua Luo, Hua Xu

**Affiliations:** Department of Thyroid, Head and Neck Surgery, Jiangxi Provincial People’s Hospital, The First Affiliated Hospital of Nanchang Medical College, Nanchang, China

**Keywords:** repair, retrospective study, surgical incision, thyroid cancer, thyroidectomy

## Abstract

**Objective:**

This study introduces a unilateral thyroid cancer resection technique using a lateral small incision via the linea alba cervicalis (the cervical midline) approach. We evaluated the differences and advantages of this method compared to the conventional low-arc-shaped cervical midline approach, focusing on minimizing surgical trauma and improving cosmetic results.

**Methods:**

Clinical data from 124 patients undergoing radical surgery for unilateral thyroid cancer at the First Affiliated Hospital of Nanchang Medical College between August 2024 and October 2025 were analyzed. Patients were categorized into either a lateral mini-incision via cervical midline approach group or a traditional low collar incision via cervical midline approach group according to the surgical approach used. A multi-dimensional evaluation compared the groups' clinical characteristics, perioperative parameters, postoperative complications, pain levels, incision characteristics, and scar cosmesis.

**Results:**

The clinical characteristics of both groups were largely comparable. The incision length in the small incision group was significantly shorter than in the traditional group (*P* < 0.05). Although operative duration and intraoperative blood loss showed no significant differences, the small incision group demonstrated significantly better outcomes for 24 h postoperative drainage volume and hospitalization costs (*P* < 0.05). Postoperatively, patients in the small incision group reported significantly lower pain scores (*P* < 0.05), higher satisfaction with incision aesthetics and scar appearance, and improved functional recovery of cervical movement (*P* < 0.05).

**Conclusion:**

The data validate that unilateral thyroid cancer resection via the cervical lateral small incision combined with cervical midline approach can serve as an alternative to traditional thyroid cancer resection surgery, while reducing surgical trauma and enhancing cosmetic outcomes.

## Introduction

Thyroid cancer (TC) is the most common malignant tumors of the endocrine system, with its incidence showing an increasing trend in recent years. Thyroid carcinoma typically originates from the epithelial cells of the thyroid gland and can be classified into various subtypes, including papillary thyroid carcinoma (PTC), follicular thyroid carcinoma (FTC), and anaplastic thyroid carcinoma (ATC). Among these, papillary thyroid carcinoma and follicular thyroid carcinoma are the most common types, accounting for approximately 80% to 90% of all thyroid carcinomas (1). The development of thyroid carcinoma is associated with multiple factors such as environmental influences, genetics, and hormone levels, particularly among high-risk populations including individuals with family histories and patients exposed to radiation. Epidemiological data show that the prevalence rate among female patients is significantly higher than that among males, typically occurring between 40 and 60 years old (2, 3). The clinical manifestations of thyroid cancer are diverse. Early stages may present no obvious symptoms, but as the disease progresses, symptoms such as lumps, a sense of pressure, hoarseness, and dysphagia may appear. Although surgery remains the cornerstone of thyroid cancer treatment, with the evolution of clinical concepts, patients have increasing expectations regarding tumor ablation efficacy, functional preservation, and cosmetic outcomes. And this has prompted a shift in treatment approaches toward comprehensive models that prioritize both functional and aesthetic outcomes in the anterior cervical region (4, 5).

Patients undergoing conventional open thyroidectomy (COT) often experience significant functional impairment in the anterior cervical region, manifesting as neck tightness and foreign body sensation. Additionally, the large midline incision compromises aesthetic outcomes. Although endoscopic approaches achieve a scar-free neck appearance, their long operative tunnels frequently lead to postoperative neck discomfort, swallowing difficulties, and sensory disturbances (6). Thus, since the 1960s, scholars have begun to attempt various lateral cervical approaches, such as the lateral cervical incision via the sternocleidomastoid muscle intermuscular space, transaxillary endoscopic thyroidectomy (TAET) (7), and endoscopic thyroidectomy via the breast-areola approach (ETBAA) (8). Compared with the traditional midline cervical incision, these approaches can better conceal the incision, yield favorable postoperative cosmetic outcomes, and provide some preservation of sensory and motor functions in the anterior cervical region. Meanwhile, traditional thyroidectomy remains dominant due to its broader indications and reliable oncological efficacy (9). However, the iterative innovations in minimally invasive thyroidectomy responding to patient needs remain a driving force for the refinement and optimization of surgical techniques.

To address this, our hospital has developed a unilateral thyroid cancer surgery technique using a lateral small incision through the linea alba cervicalis (the cervical midline) approach. This technique was compared with the traditional low-arc-shaped incision cervical midline approach thyroidectomy. A retrospective analysis was conducted to compare the feasibility and safety of the two methods (in terms of reducing surgical trauma and improving postoperative cosmetic effects, etc.).

## Methods

### Research protocol

**Figure 1:**
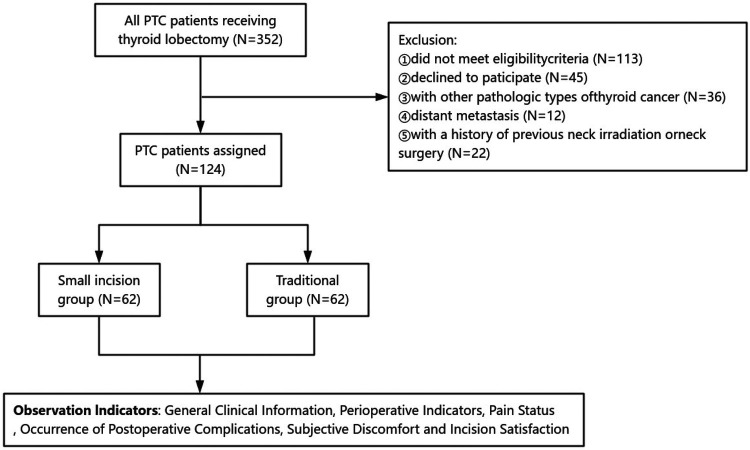
Flowchart of patients with PTC receiving thyroid lobectomy in the study. PTC, rpapillary thyroid carcinoma.

A comprehensive retrospective analysis was performed on 124 patients who underwent radical surgery for unilateral thyroid cancer at the First Affiliated Hospital of Nanchang Medical College from August 2024 to October 2025. These included 62 cases using the lateral mini-incision via cervical midline approach (small incision group) and 62 cases using the traditional low collar incision via cervical midline approach (traditional group) (Figure 1). All surgeries were performed by the same surgeon.

### Case inclusion criteria

1. Thyroid color ultrasound suggests malignant thyroid tumor with maximum tumor diameter <4 cm.
2. Fine-needle aspiration biopsy indicates thyroid papillary carcinoma.
3. Tumor located in unilateral thyroid lobe, with imaging studies showing no invasion of surrounding tissues.
4. All patients provided informed consent and have signed consent forms.

### Exclusion criteria

1. Thyroid tumor involving surrounding tissues, such as strap muscles, nerves, trachea, and esophagus.
2. Bilateral thyroid tumors requiring total thyroidectomy.
3. Poor cardiopulmonary function, impaired coagulation function, or other contraindications rendering the patient unfit for surgery.
4. Preoperative evidence of either central or lateral cervical lymph node metastasis based on imaging or cytology.
5. History of previous neck surgery or radiotherapy.

### Surgical technique overview

#### Lateral mini-incision via cervical midline approach (mini-incision group)

**Figure 2:**
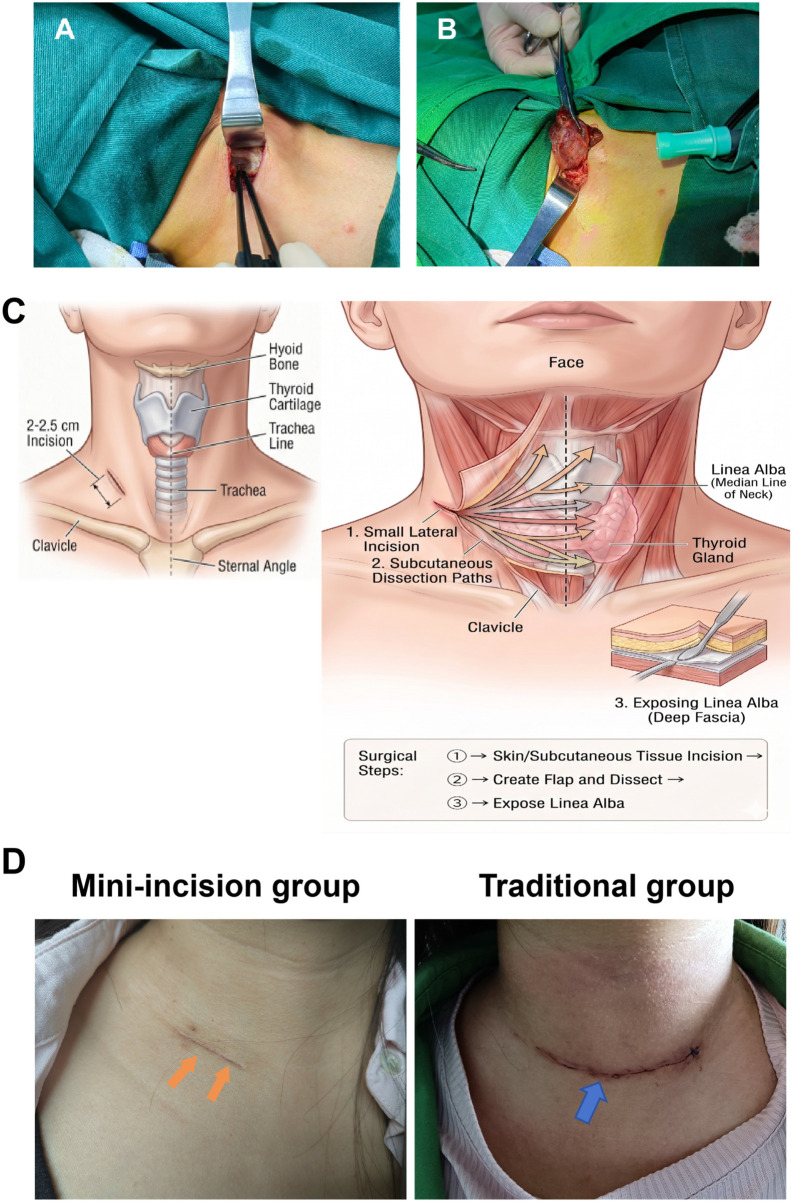
Lateral mini-incision via cervical midline approach. **(A)** Exposure of external branch of superior laryngeal; **(B)** Thyroid tumor resection; **(C)** A schematic diagram showing the subcutaneous dissection path and access to the cervical midline via the lateral mini-incision approach; **(D)** Comparison of incisions for different surgical methods.

After successful general anesthesia, patients were placed in a supine position with a shoulder pad. An approximately 2–2.5 cm incision was made above the clavicle on the affected side. The skin, subcutaneous tissue, and platysma were incised. With the help of headlight illumination, a sharp dissection was performed between the deep layer of the sternocleidomastoid muscle and the superficial layer of the anterior cervical fascia, forming a tunnel starting from the upper part of the affected side's clavicle, crossing the midline of the neck and reaching the opposite side. The assistant used the thyroid retractor to lift the flap upwards, fully exposing this tunnel. The purpose of this anatomical step was to utilize the natural loose space in the deep layer of the sternocleidomastoid muscle to reach the midline structure without tension through a lateral incision. This was a superficial surgical approach. After establishing the superficial tunnel, the deeper anatomical layers were approached. At the midline of the neck, a longitudinal incision was made through the cervical midline, entering the pretracheal space between the true and false capsules of the thyroid gland. Subsequently, the dissection was performed deep to the sternothyroid muscle and the sternothyroid muscle on the affected side, lifting this group of muscles completely off the surface of the thyroid gland, thereby fully exposing the thyroid gland on the affected side. This step was the standard cervical midline approach, used to handle the deep glands and important structures. The ablation electrode was used to divide the thyroid isthmus and inferior thyroid vein. Starting from the lateral aspect of the thyroid gland, the superior pole vessels were divided close to the upper pole while preserving the superior parathyroid gland with *in-situ* blood supply. After dividing the middle thyroid vein, the thyroid gland was retracted to the contralateral side. The recurrent laryngeal nerve was identified and fully dissected along its course to the entry point into the larynx, with care taken to protect the nerve and preserve the inferior parathyroid gland with *in-situ* blood supply. The affected thyroid gland was completely excised. Resect the prelaryngeal lympho-fatty tissues. Open the pretracheal fascia along the affected side of the trachea, completely mobilize and preserve the thymus and inferior parathyroid glands, fully exposethe recurrent laryngeal nerve, dissect the level VI lympho-fatty tissues, and harvest the paratracheal lympho-fatty tissues after identifying the entry point of the recurrent laryngeal nerve into the larynx. After checking the surgical field and achieving hemostasis, place a drainage tube in the paratracheal and paraesophageal regions, then close the incision (Figure 2).

Postoperative management: A percutaneous endotracheal esophageal side passive drainage tube was placed for all patients after surgery. The volume and characteristics of the drainage were recorded daily. The standard for removing the drainage tube was: the 24 h drainage volume was less than 15 mL, and the drainage fluid was clear, without active bleeding or chylous leakage. If the drainage volume remained above this threshold, the retention time was extended and the reason was recorded. The discharge criteria for patients was: after the drainage tube was removed, observation for at least 12 h showed no neck swelling, breathing difficulties, or abnormal incision, and the patient was able to tolerate oral diet.

#### Traditional low collar incision via cervical midline approach (traditional group)

After successful general anesthesia, patients were placed in a supine position with shoulder pad support. A 5–6 cm curvilinear incision was made along the cervical midline, approximately 1–2 cm above the sternal notch. Skin flaps were dissected bilaterally to the anterior borders of the sternocleidomastoid muscles, superiorly to the level of the thyroid cartilage and inferiorly to the suprasternal fossa. The flaps were retracted upward, followed by incision of the cervical midline. The sternothyroid and sternohyoid muscles on the affected side were separated to expose the ipsilateral thyroid lobe. The thyroid isthmus and inferior thyroid veins were divided using an ablation electrode. Subsequently, the superior thyroid vessels were ligated adjacent to the upper pole while preserving the superior parathyroid gland with intact blood supply *in situ*. After division of the middle thyroid vein, the thyroid was retracted medially to identify the recurrent laryngeal nerve, which was fully exposed and dissected along its entire course to its laryngeal entry point. Care should be taken to preserve the posteriorly located recurrent laryngeal nerve and the inferior parathyroid gland with intact vascular supply. The affected thyroid lobe was then completely resected. After resecting the prelaryngeal lympho-adipose tissues, the pretracheal fascia was opened along the ipsilateral trachea. The thymus and inferior parathyroid glands were completely dissected and preserved. The recurrent laryngeal nerve was fully exposed prior to performing Level VI lymph node dissection. Following exploration of the nerve entry point into the larynx, the paratracheal lympho-adipose tissues were excised. The surgical field was inspected and hemostasis achieved. A drainage tube was placed alongside the tracheoesophageal groove before wound closed (Figure 2).

Postoperative management: Consistent with the above group.

### Observation indicators

1. General clinical information: Age, gender, tumor location, tumor size, number of central lymph nodes dissected, etc.
2. Perioperative indicators: Including incision length, operative duration, intraoperative blood loss, postoperative 24 h drainage volume, length of hospital stay, and costs.
3. Pain status: Visual Analog Scale (VAS) (10–12) was used to assess patients'pain intensity at 24 h and 48 h postoperatively. Specifically, a Visual Analog Scale (VAS) score of 0 indicates no pain; scores of 1–2 indicate mild pain; scores of 3–4 indicate mild tolerable pain; scores of 5–6 indicate pain that affects sleep but remains tolerable; scores of 7–8 indicate intolerable severe pain accompanied by loss of appetite and sleep disorders; scores of 9–10 indicate excruciating pain that may even cause crying.
4. Postoperative complications: including incidence of postoperative hoarseness, postoperative hypocalcemia, incision infection rate, and unplanned reoperation rate.
5. Subjective Discomfort and Incision Satisfaction (13, 14) Within 3 months postoperatively, patients'subjective discomfort symptoms were recorded through outpatient follow-up. The patient scar assessment questionnaire: used to evaluate “incision satisfaction” (very satisfied, moderately satisfied, generally satisfied, dissatisfied) and “aesthetic satisfaction” (very satisfied, moderately satisfied, generally satisfied, dissatisfied). Self-designed standardized sensory questionnaire: used to assess “anterior neck sensation” (neck foreign body sensation, swallowing pulling sensation, neck constriction sensation, incision numbness sensation, no abnormalities). All subjective outcomes were independently self-assessed by the patients themselves to avoid researcher-induced bias. The assessors were independent research nurses who were blinded to the group allocation. This nurse did not participate in the surgery or postoperative clinical management, but was only responsible for distributing the questionnaires at the 3-month outpatient follow-up and collecting them on the spot. The patients had given informed consent before filling out the forms and were informed that their assessment results would not affect the subsequent treatment. Due to the objective inability to blind the patients to the incision location, this study was a single-blind design, that is, the assessors and statistical analysts were blinded to the group allocation.

### Statistical analysis

Analyses were performed using SPSS 26.0 software. Normally distributed measurement data are expressed as mean ± standard deviation (X ± S), and inter-group comparisons were conducted using independent samples *t*-tests. Non-normally distributed data were analyzed using the Mann–Whitney *U*-test. Categorical data are presented as frequencies or percentages (%), with inter-group comparisons performed using chi-square tests. Statistical significance was set at a *P*-value < 0.05.

## Results

### Characteristics of the study cohort

This study enrolled 124 patients diagnosed with PTC, including 62 cases in the small incision group and 62 cases in the traditional group. The mean age of patients was 40.52 ± 10.98 years in the mini-incision group and 46.79 ± 11.24 years in the traditional group. The mean tumor diameter was 0.87 ± 0.47 cm in the mini-incision group and 0.87 ± 0.53 cm in the c. Detailed characteristic data of all enrolled patients are shown in Table 1.

**Table 1:** Characteristics of all enrolled patients.

Items	Case	Mini-incision group (*n* = 62)	Traditional group (*n* = 62)	*P*
Age (years)	＞50	13	26	0.012
	≤50	49	36	
Gender	Male	18	10	0.086
	Female	44	52	
Tumor lacation	Left side	27	27	＞0.99
	Right side	35	35	
Tumor size (cm)	*n* ≤ 1	43	48	0.406
	1<n<2	16	10	
	2 ≤ n＜4	3	4	
Number of central lymph node dissection		3.76 ± 2.32	2.99 ± 3.21	0.632
BMI		24.66 ± 1.14	24.56 ± 1.14	0.630

### Comparison of perioperative indicators

Regarding incision size, the mini-incision group was significantly smaller than the traditional group (*P* < 0.05). In terms of operative time, the postoperative drainage volume in the mini-incision group was significantly lower than that in the traditional group (*P* < 0.05). There was no significant difference in length of hospital stay between the two groups, but hospitalization costs in the mini-incision group were significantly lower than those in the traditional group (*P* < 0.05) (Table 2).

**Table 2:** Comparison of perioperative parameters.

Items	Mini-incision group (*n* = 62)	Traditional group (*n* = 62)	*P*
Incision length (cm)	2.52 ± 0.50	5.13 ± 0.69	<0.01
Operation time (min)	46.82 ± 11.03	45.11 ± 12.23	0.268
Intraoperative blood loss (mL)	13.45 ± 9.19	16.66 ± 10.32	0.070
24 h postoperative diversion (mL)	42.23 ± 21.53	68.73 ± 28.66	<0.01
Length of stay (d)	4.82 ± 1.02	5.24 ± 1.54	0.077
Hospitalization cost (yuan)	22 824.85 ± 2 193.28	23 893.53 ± 3 099.45	0.029

### Comparison of postoperative pain scores and complication rates

A comparison of visual analogue scale (VAS) pain scores on the first postoperative day revealed significantly lower scores in the mini-incision group (2.45 ± 1.13) compared to the traditional group (2.92 ± 1.16) (*P* < 0.05). Analysis of postoperative complications across all patients showed one case of transient hypocalcemia in each group and two cases of hoarseness. No instances of incision infection or unplanned reoperation were observed. Statistical analysis indicated no significant difference in overall complication rates between the two groups (Table 3).

**Table 3:** Comparison of postoperative pain scores and complication rates.

Items	Mini-incision group (*n* = 62)	Traditional group (*n* = 62)	*P*
Incision pain	2.45 ± 1.13	2.92 ± 1.16	0.025
Postoperative hoarseness rate	0	2 (3.23%)	0.248
Postoperative hypocalcemia rate	1 (1.61%)	1 (1.61%)	0.752
Incision infection rate	0	0	—
Unplanned reoperation rate	0	0	—

### Comparison of incision satisfaction and anterior cervical region sensation

All patients completed follow-up at the third postoperative month. In the mini-incision group, incision satisfaction scores showed 45 cases (72.58%) rated as highly satisfactory, compared to 21 cases (33.87%) in the traditional group. Statistical analysis revealed a significant difference in incision satisfaction between the two groups (*P* < 0.05). Additionally, the mini-incision group reported 5 cases of cervical foreign body sensation, 3 cases of pulling sensation during swallowing, 3 cases of cervical tightness, and 4 cases of incision numbness. The traditional group showed 10 cases of cervical foreign body sensation, 7 cases of pulling sensation during swallowing, 6 cases of cervical tightness, and 7 cases of incision numbness. The comparison of cervical anterior region sensory abnormalities between groups demonstrated a significantly lower abnormality rate in the mini-incision group than in the traditional group (*P* < 0.05) (Table 4).

**Table 4:** Comparison of postoperative satisfaction, anterior cervical sensation, and aesthetic effects.

Items	Category	Mini-incision group (*n* = 62)	Traditional group (*n* = 62)	*P*
Incision satisfaction	Highly satisfactory	45 (72.58%)	21 (33.87%)	<0.01
	Moderately satisfactory	10 (16.13%)	14 (22.58%)	
	Generally satisfactory	7 (11.290%)	27 (43.55%)	
	Dissatisfied	0	0	
Anterior neck sensation	Abnormal	Foreign body sensation in neck 5, pulling sensation during swallowing 3, neck constriction 3, incision numbness 4 (24.19%)	Foreign body sensation in neck 10, pulling sensation during swallowing 7, neck constriction 6, incision numbness 7 (48.39%)	<0.01
	Normal	47 (75.81%)	32 (51.61%)	
Aesthetic satisfaction	Highly satisfactory	37 (59.68%)	12 (19.35%)	<0.01
	Moderately satisfactory	12 (19.35%)	22 (35.48%)	
	Generally satisfactory	13 (20.97%)	28 (45.16%)	
	Dissatisfied	0	0	

### Comparison of aesthetic effects

**Figure 3:**
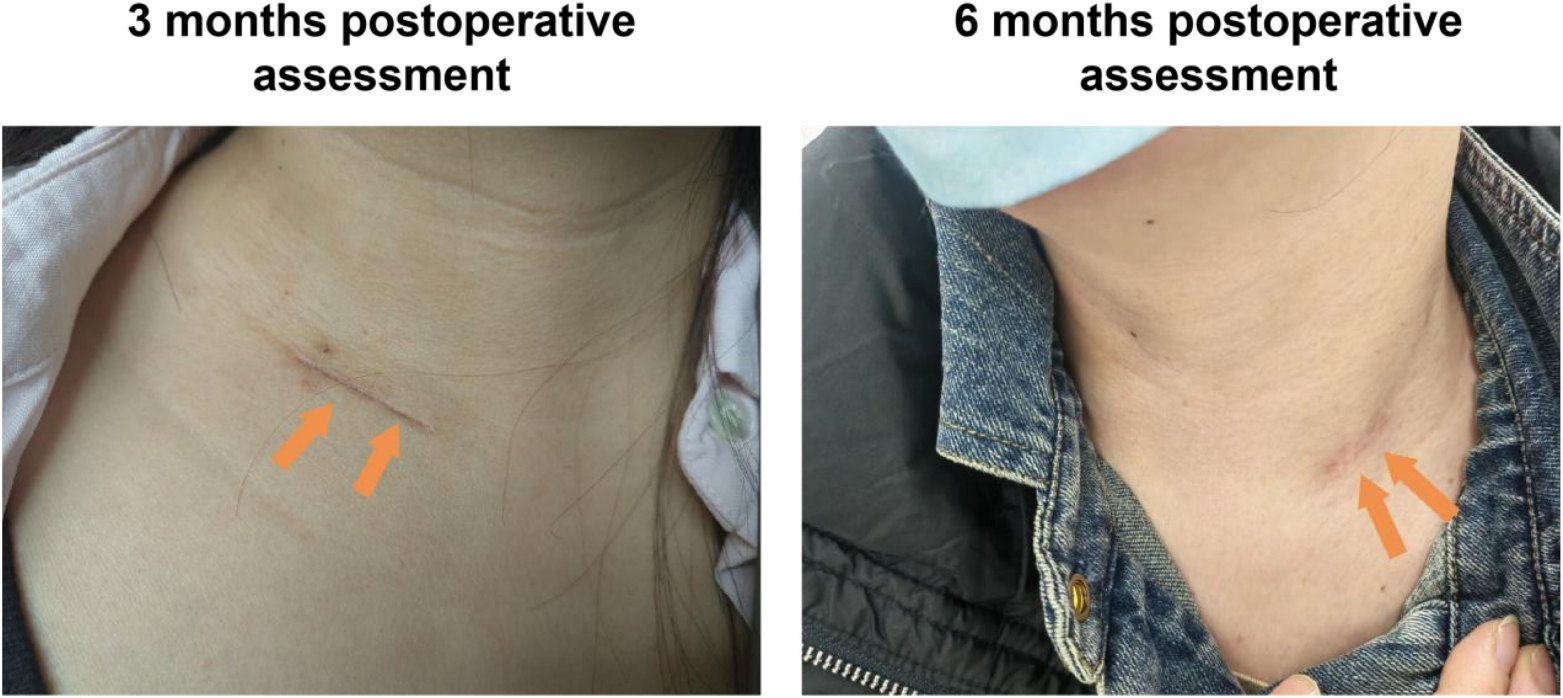
Representative images of mini-incision patients in different postoperative times.

Simultaneously, we analyzed patients' evaluations of the postoperative aesthetic effects of incisions. The results reported a significantly higher rate of highly satisfactory ratings in the mini-incision group compared to the traditional group (*P* < 0.05) (Table 4). Observation revealed significant effects in the mini-incision, including scar lightening and reduced thickness (Figure 3).

## Discussion

Traditional thyroidectomy employs a circumferential incision via the anterior cervical approach, a technique pioneered by Theodor Kocher in the late 19th century. Advances in surgical technology and rising patient demand for better cosmetic outcomes have driven thyroid surgery's evolution—from open to endoscopic-assisted, and now to robotic-assisted techniques (15). However, the problems associated with the use of endoscopes in neck surgeries cannot be ignored. These include concerns such as the large surgical area causing postoperative pain and various complications (16), the impact of postoperative facial nerve damage on patients' lives (17), the significantly longer operation time compared to traditional open surgeries, the increased rate of incision infections, and the longer learning period for surgeons and higher costs associated with this endoscopic surgical method (18–21). The lateral cervical approach surgery has become the focus of many surgeons.

The minimally invasive thyroidectomies (MIT) was firstly reported by Park et al. (22) in 2001 using a 3.0–4.5 cm cervical incision. Compared to traditional open surgery, this technique offers advantages including shorter operative time, reduced blood loss, faster postoperative pain relief, shorter length of hospital stay, and decreased drainage tube placement. Subsequently, many experienced thyroid surgeons have made exploratory attempts at thyroidectomy with shortened incisions without the need for endoscopic assistance (23–25). The supraclavicular oblique incision thyroidectomy (SOIT) is a unilateral 2–3 cm incision made along the lateral border of the clavicle. This technique has been developed over the past decade to achieve better cosmetic outcomes (26, 27). Jiang et al. (6) compared the differences in perioperative and long-term outcomes between patients undergoing traditional low-collar open thyroidectomy (TLCIT) and those receiving supraclavicular oblique incision thyroidectomy (SOIT). They found that patients who underwent SOIT had advantages such as smaller incisions, shorter postoperative drainage time, reduced drainage volume, and shorter length of hospital stay. Furthermore, it demonstrated superior outcomes in cosmetic effects and neck sensation, among other aspects. Ren et al. (28) reported that patients undergoing SOIT showed no significant differences compared to those receiving TLCIT in all indicators except incision length. However, in that study, incision lengths for both surgical approaches exceeded 5 cm. In our study, the lateral mini-incision via cervical midline approach resulted in a significantly smaller incision length than the traditional midline cervical approach (2.52 ± 0.50 vs. 5.13 ± 0.69 cm, *P* < 0.05). Additionally, this method offers advantages including lower postoperative drainage volume, quicker pain relief, and reduced hospitalization costs. Studies have shown that the use of 2.5–3 cm small incisions during surgery resulted in transient hypocalcemia in 36.6% of patients and transient vocal hoarseness in 24.4% of patients, although symptoms improved significantly during 3-month postoperative follow-up (29). In contrast, our data demonstrate superior safety outcomes, with postoperative rates of transient hypocalcemia and vocal hoarseness maintained below 5%. And it is important to note that the superior postoperative outcomes in the mini-incision group are inextricably linked to the significantly shorter incision length. The anatomical advantage of entering via the relatively avascular and neural-free midline allows for adequate surgical exposure through a 2.0–2.5 cm window. While the smaller incision itself contributes to reduced pain and faster recovery, it is the cervical linea alba approach that provides the technical possibility to safely operate through such a limited access, which is the defining clinical advantage of this technique over the conventional approach.

Overall, this retrospective analysis summarizes the key characteristics of the mini-incision approach compared to the conventional approach: 1) The incision is smaller and more concealed, located within the natural skin lines above the clavicle, with scars almost completely concealed by clothing collars. Patient satisfaction with cosmetic outcomes at 3 months postoperatively was significantly higher than with traditional low-collar incisions; 2) Access is gained through the natural plane of the linea alba without transecting strap muscles, reducing skin flap dissection area by approximately 50%. Postoperative 24 h drainage volume decreased by an average of 26.5 ml, resulting in milder tissue trauma and faster recovery; 3) The recurrent laryngeal nerve is fully exposed to its laryngeal entry point throughout the procedure, reducing transient hoarseness incidence and decreasing anterior cervical sensory impairment by over 50%. Additionally, this approach requires no specialized instruments, and its operative technique remains comparable to traditional approaches. After dedicated training, all thyroid surgeons at our institution have successfully mastered this technique. While remote-access endoscopic thyroidectomy has gained popularity for its superior cosmetic outcomes, it is often associated with more extensive internal tissue dissection and higher costs. Our results suggest that the mini-incision via the cervical midline approach serves as a viable “third option”. It offers a pragmatic balance by minimizing the external scar compared to conventional thyroidectomy without the internal trauma or technical complexity of endoscopic techniques. This approach is particularly well-suited for patients seeking a minimally invasive option that is both safe and cost-effective, especially in clinical settings where remote-access surgery may not be the first choice due to patient-specific or economic factors.

However, this study has several limitations, including a relatively small sample size, a short follow-up period, and exclusion of cases requiring total thyroidectomy for bilateral thyroid tumors. The average postoperative hospital stay in this study was approximately five days, which is longer than the ambulatory or 24 h discharge protocols reported in some literature (30). In our clinical pathway, we prioritize the prevention of postoperative hematoma. Drains are typically removed only when the output is less than 10–15 mL per 24 h, which usually occurs on postoperative day 3 or 4. Besides, to ensure patient safety and monitor for potential complications such as recurrent laryngeal nerve palsy or hypocalcemia (especially for patients traveling from rural areas), a more conservative observation period was maintained during the study period. And The postoperative length of stay is also influenced by the local healthcare system and insurance policies, where inpatient recovery is often preferred by patients to ensure they are fully stabilized before discharge. While these findings reflect our specific clinical environment, future studies under enhanced recovery after surgery protocols may demonstrate further reductions in hospitalization time. Furthermore, our study specifically selected low-risk PTC patients to evaluate the safety of the surgical approach. Future research should focus on conducting more long-term prospective studies, expanding sample sizes, and assessing the improved oncological efficacy and broader applicable scope of this method.

## Conclusion

Compared with traditional thyroid cancer resection, the unilateral thyroid cancer resection via the lateral cervical small incision combined with cervical linea alba approach yields more aesthetically pleasing incisions; provides superior protection of anterior cervical function; and maintains equivalent surgical safety and thoroughness. This approach can serve as a viable alternative to traditional surgery. The operative techniques are comparable to traditional approaches with similar difficulty levels, which facilitates widespread adoption of this novel technique.

## Data Availability

The original contributions presented in the study are included in the article/Supplementary Material, further inquiries can be directed to the corresponding author/s.
